# The design and development of a home-based rehabilitation programme for those recovering after an episode of delirium

**DOI:** 10.1186/s12913-025-13614-8

**Published:** 2025-11-12

**Authors:** Alison J. Bingham, Abigail Laverick, Kirstie Chandler, Shruti Raghuraman, Victoria A. Goodwin, Sarah Morgan-Trimmer, Lesley Collier, Rowan H. Harwood, Obioha C. Ukoumunne, Rachael Litherland, Elizabeth Goodwin, Alasdair M. J. MacLullich, Jinpil Um, Sarah J. Richardson, Linda Clare, Louise Allan

**Affiliations:** 1 Faculty of Health and Life Sciences, University of Exeter Medical School, University of Exeter, Exeter, UK; 2 Innovations in Dementia, Exeter, UK; 3 NIHR Applied Research Collaboration South West Peninsula, University of Exeter, Exeter, UK; 4 School of Health Sciences, University of Nottingham, Queens Medical Centre, Nottingham, UK; 5 Geriatric Medicine, Edinburgh Delirium Research Group, Usher Institute, University of Edinburgh, Edinburgh, UK; 6 AGE Research Group, NIHR Newcastle Biomedical Research Centre, Faculty of Medical Sciences, Newcastle University, Newcastle upon Tyne, UK; 7 Cumbria Northumberland Tyne and Wear NHS Foundation Trust, Newcastle upon Tyne, UK

**Keywords:** Delirium, Recovery, Rehabilitation, Complex intervention, Intervention development, Frailty

## Abstract

**Background:**

Delirium, closely linked to increasing age and frailty, is a growing concern in the aging population, yet there is little understanding about how to support recovery for individuals and their carers. This paper details the design and development of RecoverED, a home-based rehabilitation intervention for delirium recovery.

**Methods:**

A realist-informed approach was used to develop a programme theory and logic model for RecoverED. A rapid realist review had identified strategies for delirium recovery, followed by interviews with stakeholders (older adults, carers, and professionals), and an expert panel discussion. The intervention was then developed based on the refined programme theory of what had worked to improve recovery from delirium, for whom, and in what context.

**Results:**

The RecoverED intervention, described using the TIDieR checklist, was a complex, multicomponent, 12 week home-based programme delivered by a multidisciplinary team in up to 10 sessions. The intervention comprised cognitive, physical, and psychosocial components. An intervention manual and training programme had been developed to support delivery teams.

**Conclusion:**

The RecoverED intervention was being evaluated in a multi-centre feasibility trial with a qualitative process evaluation. This paper describes theory-based rehabilitation interventions for long-term delirium recovery. Further research through a randomised controlled trial is needed to assess its effectiveness and cost-effectiveness before broader implementation.

**Supplementary Information:**

The online version contains supplementary material available at 10.1186/s12913-025-13614-8.

## Background

Delirium, sometimes called ‘acute confusional state’, is a common clinical syndrome characterised by disturbed consciousness, cognitive function and/or perception, which has an acute onset and fluctuating course [1]. It is estimated that over 20% of hospitalised older adults will experience an episode of delirium [2], with delirium being consistently associated with increased mortality [3]. Delirium incidence is strongly associated with age, prior dementia and frailty and so in an ageing population, it is a growing problem [1]. People who develop delirium sometimes have persisting symptoms and are more likely to be re-hospitalised within 30 days, visit the emergency department, or be discharged to a location other than their own homes [4]. A delirium episode is associated with other prolonged negative outcomes such as loss of independence and poor functional recovery after discharge [2, 5–7]. Despite this, there is little rigorous evidence about how to support people with delirium and their informal carers, especially in the community setting after discharge from hospital.

To address these knowledge and practice gaps, a novel, complex multi-component rehabilitation intervention known as the RecoverED (Recovery after an Episode of Delirium) Intervention was developed. The main objective of the RecoverED intervention was to improve recovery after delirium in post-acute, community settings in older people with or without dementia. Outcomes of interest included patient-related improvements in activities of daily living, mobility, cognition, mood and wellbeing, and quality of life in addition to carer outcomes including carer burden, quality of life and wellbeing.

The development of the RecoverED intervention followed the Medical Research Council (MRC) framework for designing and evaluating complex interventions to improve health care [8]. The application of MRC guidance follows the presumption that any rehabilitation intervention focused on improving outcomes for people with delirium will reflect the complexities of the condition, with patients experiencing multiple comorbidities and presentations of their problems, and complex social context requiring a combination of approaches to achieve optimisation of outcomes.

The MRC framework delineates four phases in complex intervention research: development or identification of the intervention, feasibility, evaluation, and implementation. The development phase involves uncovering the implicit theoretical basis of an intervention and developing a programme theory [8] or theory of change. A programme theory can be represented by a logic model that connects the activities and resources of a programme to its expected outcomes. This model is then used to guide the evaluation of the programme [9]. It serves many purposes including identifying key components and the interactions between them, mechanisms of change, contextual factors and how they interact with the components and the change mechanisms, and how desired and relevant outcomes are produced and sustained.

The purpose of this paper is to systematically describe the design of the RecoverED intervention following the MRC guidance while using a multidisciplinary, patient-centred approach to development, grounded in explicit theory on how the intervention operates.

## Methods

### Programme theory and logic model

**Fig. 1:**
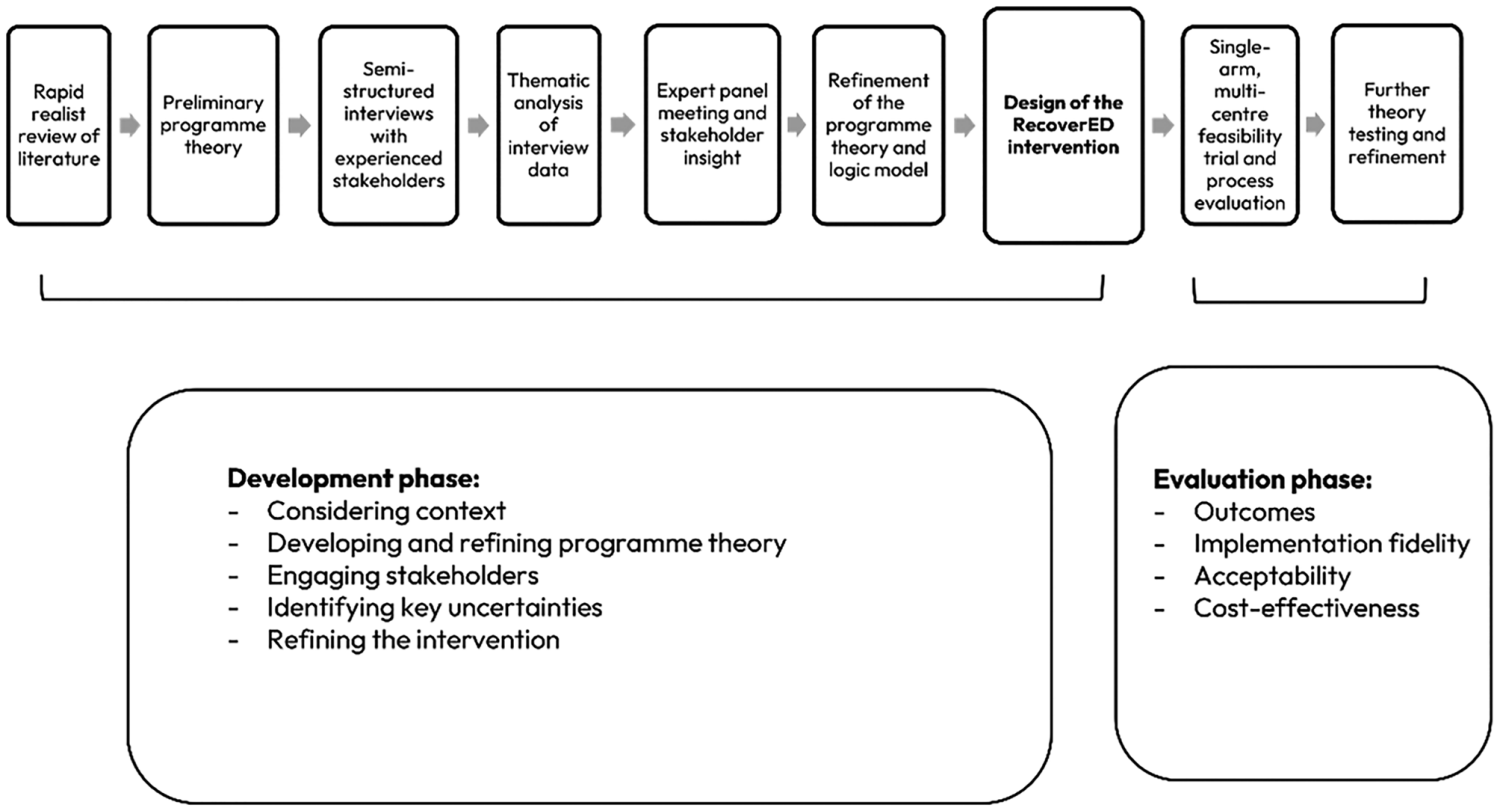
Development process of the RecoverED intervention aligned with phases of the MRC framework

A programme theory and logic model were developed. This process drew on a realist approach, to develop a theoretical understanding of what works to help improve recovery after delirium, how, for whom and in what circumstances. Realism is a philosophical approach to understanding complex social situations, identifying potential mechanisms for change and how these interact with contexts to produce outcomes [10]. The development process is presented in Fig. 1.

**Fig. 2:**
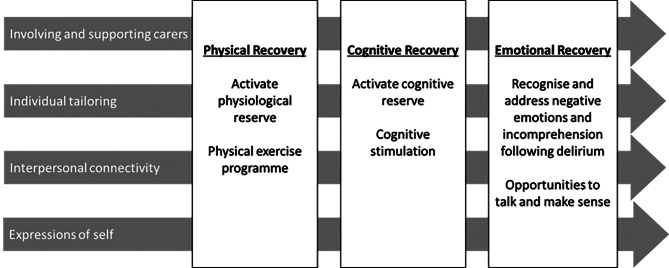
Recovery domains and facilitators (O’Rourke et al., 2020)

In the first stage, a rapid realist review of literature was undertaken to identify strategies that might improve recovery from delirium, how, why, in what circumstances and for whom [11]. A preliminary programme theory was developed following a synthesis of 52 studies, outlining three interrelated recovery domains: physical, cognitive and emotional. The theory statements for each domain are presented in Fig. 2.

The authors stressed the interdependence of each recovery domain, and highlighted evidence to support the value of multicomponent interventions in supporting global recovery of people who had had delirium.

In the next stage, in-depth interviews were conducted with key stakeholders including older people who had experienced an episode of delirium, their informal carers, and health and social care professionals with expertise in delirium care. Forty-six people were interviewed in total, (8 older people who had experienced delirium, 14 carers, and 24 health and social care professionals). The data were analysed using inductive and deductive thematic analysis and realist analysis to refine the preliminary programme theory. A panel of experts consisting of researchers, medical professionals, health and social care professionals, and patient and public involvement representatives were given an opportunity to offer views on the findings and the preliminary programme theory. Based on their insights the programme theory was further refined [12], and a logic model was developed and is presented below in summary form in Table 1.

**Table 1:** RecoverED Intervention – Summary of the logic model

Core components	Mechanism responses	Short-term impacts	Impacts	Outcomes
Physical Recovery	Support and access to planned, structured and repetitive strength and balance exercises, functional activity practice, confidence building exercises	Recovery and maintenance of physical function, improved functional ability (activity and participation), reduced excess disability	Improved physical health, reduced frailty, better functional ability, delay of disability	Improved physical health, better mobility and reduced frailty, maintenance of independent living skills
Cognitive Recovery	Support with and access to goal-oriented activities aimed at improvements in attention, executive function, memory, guided practice of selected everyday activities, use of enhanced learning and compensatory strategies	Protection against further/rapid cognitive decline, improved functional ability (activity and participation), reduced excess disability	Recovery of cognitive abilities, better functional ability in targeted activities	Improved cognition, delay in cognitive decline, maintenance of independent living skills
Psychosocial Recovery	Access to skilled professionals jointly with patient and carer to address any distress, low mood, anxiety symptoms. Identification of barriers to social contact, motivation and encouragement to safely increase social contact and interaction	Sense-making, fewer mood and anxiety symptoms, reduced distress, greater social engagement	Better psychological state, access to social networks, better functional ability in targeted activities	Better mental health, improved wellbeing and better quality of life
Delirium Education	Access to information about delirium, its care, what to expect.	A better understanding of delirium, improved confidence in managing symptoms for both patients and carers, more involvement with care planning	Reduced fear and anxiety, better psychological state	Improved patient- and carer-well being
Monitoring active treatment and signposting	Support with coordinating with medical services, allied health services	Improved physical/physiological health	Fewer delirium symptoms, better health, better medical management	Better recovery from delirium, improved health, preventing re-hospitalisation or care home placement
Healthy lifestyle guidance	Awareness about sleep hygiene, optimal nutrition and hydration, safety netting	Better sleep practices, improved diet and water intake, conducive physical environment for improved recovery readiness & success, rebuilding daily routines	Fewer delirium symptoms, better health	

The programme theory identified six interrelated components that were relevant to supporting recovery after being discharged from hospital following an episode of delirium in older people. These included activities to support physical, cognitive and psychosocial recovery, delirium education, monitoring of active treatment and healthy lifestyle guidance. The RecoverED intervention was designed so that each individual would receive a structured, yet tailored and flexible programme that took their needs, goals, and abilities into account. This approach, including tailoring and carer involvement, was considered in every aspect of intervention design and delivery.

The next stage was the design of the RecoverED intervention based on the refined programme theory and logic model. This paper primarily describes the design of the intervention.

### Collaborative working and stakeholder engagement

The intervention was drafted collaboratively by a physiotherapist (PT), an occupational therapist (OT) and a clinical psychologist (CP) with clinical experience in dementia and delirium care, based on the programme theory. The programme design was also informed by the principles of the International Classification of Functioning, Disability and Health (ICF) [13]. The ICF offers a holistic view of health and provides a shared language which helps with communication across professions [14]. The intervention was then refined in consultation with a wider team consisting of a senior geriatrician, an occupational therapist, a physiotherapist and a clinical psychologist with expertise in ageing and neurocognitive disorders. The individual components were designed to complement each other and work together to create a cohesive intervention. The complexity of the patient group, and of delirium as a condition, necessitated multidisciplinary team collaboration in intervention development. Each professional brought their own clinical knowledge and experience and was guided by the programme theory and logic model. The existing evidence base for each individual discipline was used to design each component of the intervention, including development of participant materials and the manual.

### Patient and public involvement and engagement (PPIE)

Patient and public involvement and engagement (PPIE) was utilised to support design and development of the intervention. Facilitation was provided by a PPIE collaborator from Innovations in Dementia, a not-for-profit community interest company (CIC) that can liaise between organisations and people with dementia to shape practice, policy and attitudes. A group of patients and their carers was involved in the design of the intervention to ensure that it was practical, patient-centred, culturally relevant and tailored to the needs of the target population. While the majority of the PPIE group identified as white, two members identified as non-white, contributing valuable perspectives to the development process. Feedback from the PPIE group played a role in shaping the Recovery Record, an educational resource for participants that included the delirium information sheet. A member of the PPIE group assisted with the production of resources for the physical recovery component of the intervention, including photograph sheets for the individual activity sheets. They acted as models for the photographs and gave insights to assist with co-designing this part of the intervention.

## Results

The RecoverED intervention was described following the Template for Intervention Description and Replication (TIDieR) checklist [15], starting with a description of the intervention components and the practical steps involved in delivering the intervention. Details regarding implementation strategies were described as the programme theory identified them as an important aspect of delivery. These include training modules, a delivery manual and the additional supporting materials, as well as supervision and support elements.

The RecoverED intervention was delivered in individuals’ own homes following discharge from an acute hospital admission. This included supported living arrangements but excluded residents of nursing or residential care facilities. The programme focused on those who had received a clinical diagnosis of delirium whilst in hospital, diagnosed using clinical criteria or well-established screening tools including the Confusion Assessment Method or the 4AT Rapid Clinical Test for Delirium Detection [3]. Continued symptoms of delirium post hospital discharge were not an eligibility criterion.

### Core intervention components

#### Physical recovery

The programme theory reflected the need for physical rehabilitation to be planned, structured and repetitive, with a focus on strength and balance exercises. Integrating intervention activities into the everyday lives, activities, and routines of persons with delirium was identified to optimise effectiveness. However, given the cognitive, attentional, and motivational deficits that commonly accompany delirium and its recovery, a tailored approach was necessary to ensure engagement and sustained participation. This included simplifying instructions, using visual prompts or demonstrations, breaking tasks into manageable steps, and allowing additional time for task completion.

A selection of strength and balance activities was chosen for use in RecoverED, organised into three levels, with the degree of challenge increasing across the levels. PTs selected the most suitable activities based on findings from the initial assessment and in alignment with the person’s individual goals and cognitive capacity. The selected movements were incorporated into daily life and were designed to complement chosen activities in other recovery domains, a similar approach to that used in the Lifestyle-Integrated Functional Exercise (LiFE) programme [16].

Implementation of exercise in daily life (adherence) required consideration of behaviour change frameworks [17]. Approaches which fell under education, modelling and persuasion as outlined by the Behaviour Change Wheel [18], were employed to encourage habitual change within the physical recovery component of RecoverED. These techniques were tailored to the individual’s needs, taking into account potential memory difficulties, reduced initiation, and fluctuating attention common in this population.

#### Cognitive recovery

Cognitive recovery was addressed in several ways through the RecoverED intervention. The programme theory suggested that focusing on function in everyday tasks which require a range of cognitive skills was likely to be key to overall cognitive recovery. To reflect this, the intervention was designed to support engagement in graded and personalised activities of daily living (ADL). This approach aimed to make improvements in cognitive performance generalisable beyond the intervention specific task by equipping the participants with transferable skills.

Given the cognitive challenges typically experienced following delirium, including impairments in attention, memory, executive functioning, and motivation, the intervention incorporated adaptations to maximise accessibility and engagement. These included simplifying instructions, using repetition and structured routines, breaking down complex tasks into smaller, achievable steps, and allowing flexible pacing to accommodate cognitive fatigue or fluctuating attention.

Enhanced learning techniques including, expanding rehearsal, errorless learning and effortful processing [19] were embedded throughout the intervention to support the acquisition and retention of skills, with opportunities for both learning and re-learning integrated into everyday activities. A combination of these strategies was considered necessary to meet the diverse recovery needs of the target population, particularly in light of the varied cognitive profiles and rates of recovery following delirium.

To support the design of a personalised and achievable intervention ADL function was assessed using an adapted version of the Pool Activity Level (PAL) Checklist [20]. This tool invites individuals to rate examples of practical ADL tasks that require a range of cognitive skills including attention, memory skills and executive functioning. Originally designed for use in nursing home settings, the PAL checklist was adapted to reflect the intervention’s delivery in the participants home, including the possibility that some participants may be living alone. The assessment was completed collaboratively by the OT with the older adult/carer dyad.

Once the PAL assessment was complete and the participant’s activity level identified (see Supplementary file 3 for examples of guidance on each activity level) a personalised goal was developed collaboratively between the delivery team and the participants. The OT was then guided to create a personalised, task-oriented intervention that met the cognitive rehabilitation needs of the patient and carer participants.

In addition to the specific ADL focused cognitive recovery intervention participants were offered orientation activities and lifestyle advice, via the recovery record, with a focus on supporting memory function and encouraging engagement in meaningful occupation. Information was provided on how to utilise enhanced learning techniques [19] for those aiming to address memory impairment. Compensatory strategies were also recommended depending on the individual’s level of functioning, individual preferences and overall rehabilitation goals.

#### Psychosocial recovery

From the initial literature review and interviews with people who had experienced delirium and carers, the social and emotional consequences of delirium emerged as an important recovery domain and psychosocial support formed a key element of the intervention logic model.

Supporting people to reconnect with family and social networks was expected to contribute to improving emotional well-being. Facilitating social reintegration was a foundational element throughout the intervention, with the sessions providing an experience of social interaction that could be built upon and extended to other social and everyday activities. Barriers to socialising, whether internal such as lack of confidence or external such as lack of access to transport, could be identified and addressed during sessions, and signposting to relevant community groups or activities used to encourage increased social contact.

Alongside social reintegration, the emotional impact of delirium covers a wide spectrum, potentially resulting in symptoms of post-traumatic stress disorder, and often requires more focused psychological support. The challenge was in developing opportunities for emotionally focused recovery and psychologically informed support that could be safely delivered by trained Rehabilitation Support Workers (RSW) and their professionally registered OT and PT supervisors. There were few clinical practice guidelines available to direct development of interventions targeting post delirium emotional sequelae including symptoms of depression, anxiety, and trauma [21], and there was some concern that offering psychologically informed interventions without supervision from a registered psychologist could potentially increase risk of self-harm and suicide. To address this, a toolkit of psychological interventions and a set of guidelines were developed to enable RSWs to identify signs suggesting a need to refer the person for more specialist help and support and to take appropriate action over any concerns about suicidal thoughts or intent.

Three simple and open ‘mood and feelings’ questions were asked at initial assessment and every intervention session. These were intended to hold open the space for psychosocially focused active listening by the intervention teams, based on a trauma-informed approach that emphasises building trust, creating a sense of safety, empowering the person, collaborating in setting goals, and offering choice. Depending on the responses and on their observations of the person, RSWs could then collaboratively select from coping strategies or exercises designed to address worry, low mood or depression, stress and trauma.

#### Delirium education

The programme theory identified the need for the provision of information and signposting to resources about delirium, and to normalise and legitimise person’s/carer’s responses to coping with delirium. An information sheet, designed with input from the PPIE group, featured within the Recovery Record. It provided details about the causes, symptoms and management of delirium. Two delirium information video resources were also created with PPIE members. These involved informal discussion about the lived experiences of a carer and the experiences of those who had experienced delirium with the aim of helping with sense-making and normalising the situation for those affected.

#### Healthy lifestyle guidance

Healthy lifestyle guidance was recognised as a key element in supporting recovery and reducing the risk of future episodes of delirium. This guidance was included in the Recovery Record and covered strategies for optimising the environment and daily routines to promote healthy sleep habits. An information sheet was also provided, outlining the importance of a balanced diet and offering advice on where to access additional nutritional support.

#### Monitoring active treatment and signposting

This component also featured in the Recovery Record; delivery teams were encouraged to discuss both physical and mental wellbeing at each intervention session. A list of prompt questions about general physical health as well as mood and feelings were provided to guide these conversations. The aim of this was introducing early intervention if the person’s health deteriorated. This means that timely onward referral or signposting for unmet needs for either member of the dyad could then be made.

### Intervention delivery

**Fig. 3:**
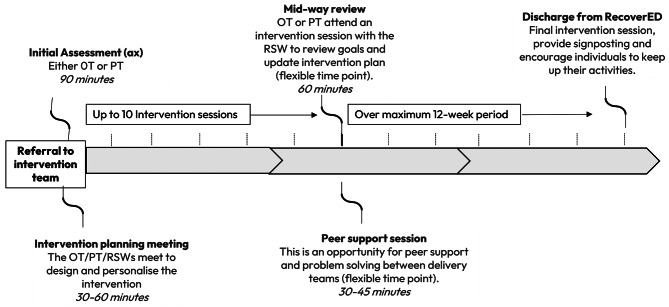
Intervention delivery process

The intervention was delivered in up to 10 personalised sessions over 12 weeks, all within the individual’s home or local area. Ten sessions were anticipated to be a sufficient ‘dosage’ to meet the participant pair’s needs, and also an acceptable length of time to be considered for adoption into usual care pathways. Sessions were delivered by a combination of PT, OT, and RSW, depending on the specific needs of the participant and the service context. Figure 3 illustrates the key milestones of intervention delivery.

#### Initial assessment

Following discharge from hospital, both the person and their carer were present at the initial assessment visit. The carer was required to be a friend or family member who had at least one hour of contact with the person each week. While they were not required to live with the person, their attendance at the initial assessment was mandatory. Attendance at subsequent visits was encouraged but not required. The OT or PT documented the individual’s current abilities, impairments, and personal goals to facilitate tailoring of the intervention. Physical function was measured through the timed up and go assessment, paired with observation and information about premorbid function. The adapted PAL [20] was used to guide the therapist to the level at which the person is able to engage in daily activities. Emotional wellbeing was assessed through open-ended questions designed to prompt reflection on how delirium had disrupted daily routines and impacted emotional health.

#### Individualised goal setting

The intervention used goal setting as a basis to tailor the intervention. Activities were selected in accordance with the needs, wishes, and mutually agreed goals of the person and, where appropriate, the carer. An overarching participation goal was established, which was then broken down with support from the therapy team into Specific, Measurable, Achievable, Realistic, Time-based (SMART) goals. This meant that no two people received the same intervention. The number, frequency and length of intervention sessions varied based on individual preference and need. Goals were revisited at each session, and progress monitored by the delivery team. Goals were formally reviewed at the mid-way review session and at the end of the intervention. However, changes could be made at any stage by a registered therapist following discussion with the person, their carer, and the RSW.

#### Planning the intervention

Once the initial assessment was completed, the assessing therapist planned an individualised intervention in collaboration with the wider team (OT, PT, and RSW), based on the agreed SMART goal(s). There were individual instructions for designing interventions associated with each of the main intervention components.

**Fig. 4:**
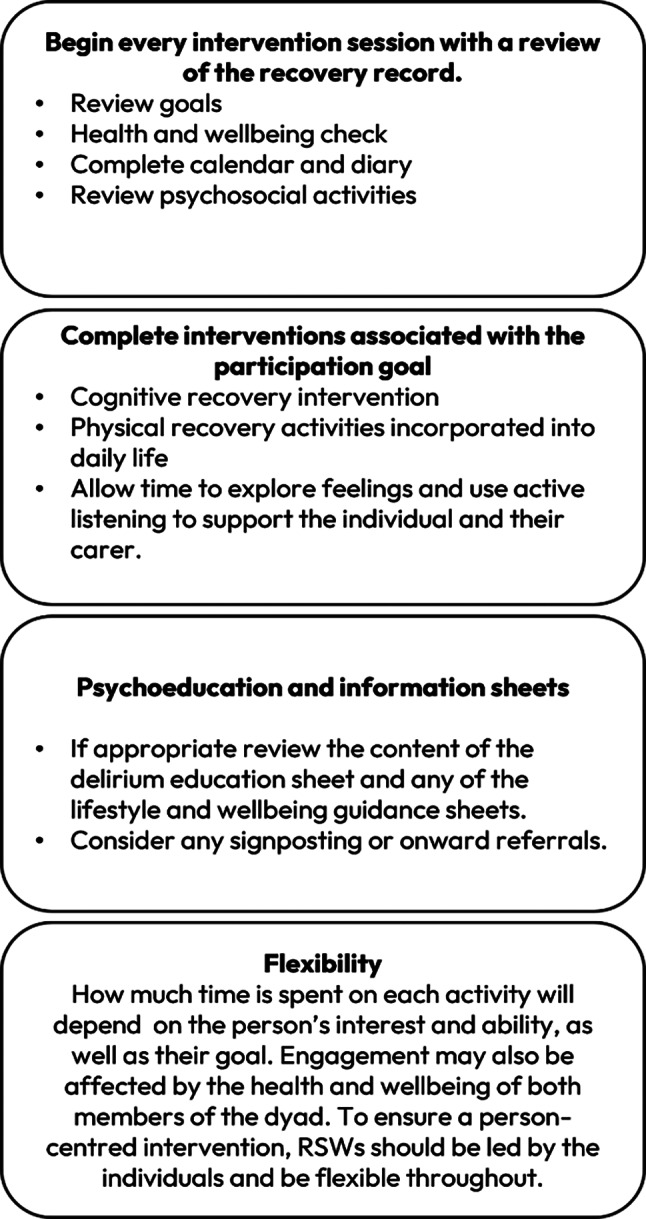
Intervention session structure

Guidance on structuring intervention sessions was provided. Figure 4 shows the recommended structure of each intervention session, whilst highlighting that the sessions are flexible and tailored so not every session takes exactly this format.

#### Midway review

A flexible approach to reviewing goals was taken throughout the intervention with a formal midway review session, led by a registered therapist, built into the structure of the programme. This could have occurred at any point during the programme and ensured the person’s goals and the structure of the intervention remained relevant and achievable. Changes to goals could have been made at any stage, or new goals added. The intervention design was responsive to changing need so activities could have been added, modified, or removed at any stage.

#### Concluding the intervention

The intervention was designed to end at a time suited to the person and their carer, with a maximum of ten sessions available. If after ten sessions the individual had ongoing rehabilitation needs, the delivery team were encouraged to explore, in discussion with the person and their carer, options for onward referral to local services.

### Implementation strategies

#### Training manual and materials

A comprehensive training package was produced to support all staff involved in intervention delivery. Prior to starting the training each person was given a pre-reading document featuring delirium education and a section which asked them to consider and note down what services are available in the intervention team’s local area. The training modules consisted of a series of presentations, accessible online for clinicians and RSWs to complete at their own pace. These were designed not only to enhance knowledge but also to foster confidence among staff in delivering the tailored intervention. Supplementary file 1 includes a table which shows the content of the modules.

The intervention was manualised, and the manual was split into two sections. The first section detailed the theoretical background of the intervention, including information about the condition, typical treatment pathways, and how the RecoverED study addressed gaps in the literature. The second section of the manual provided a detailed guide to delivering the intervention, including all resources and documents needed to design and implement a tailored, personalised intervention, regardless of delirium symptoms and the complexity of individuals’ health and social care needs. The manual covered:

1. Background information about delirium and recovery from delirium.
2. An overview of the RecoverED study and how it was developed.
3. The roles and responsibilities of the people and organisations involved in RecoverED.
4. Information about training and instructions on how to deliver the intervention, including paperwork to guide the intervention design.
5. The role of each staff member involved in providing feedback on the intervention.

Accompanying the manual was the Recovery Record, a booklet containing information and resources for the person and carer. Details of the contents of the Recovery Record and how it is intended to be used as a part of the multi-component intervention are in Supplementary file 2.

#### Support and supervision

Intervention delivery support strategies were developed, outlined in Table 2.

**Table 2:** Support and supervision strategies

Strategy	Aims
Peer support session	1. An opportunity to ask questions of the study team 2. An opportunity for peer support, to share experiences, and to learn from others delivering the intervention. 3. A chance to give feedback to the research team to help with future refinements.
Ad hoc supervision	Members of the study team were available throughout delivery for ad hoc support, which could be provided via email, telephone, or video call. This was needed as the intervention was novel.
Supervision	Provided to the RSW by the therapists within the delivery team. The RSW should seek support and advice from the relevant OT or PT as required throughout intervention delivery.

## Discussion

This paper has described the design and development process of the RecoverED intervention, which is a multi-component intervention intended to maximise recovery following an episode of delirium, developed using the MRC framework for intervention development. The intervention built on previous stages of development, utilising the findings of a realist review of existing rehabilitation approaches and services, a qualitative study and findings from engaging key stakeholders and experts. The intervention aimed to support people over the age of 65 to recover after an episode of delirium during hospital admission. Individuals were at different stages of recovery at the point of discharge from hospital. The novel multi-disciplinary approach to the intervention development brought together the skills and experience of a range of professionals resulting in a diverse and holistic rehabilitation intervention.

The strength of this intervention development process lay in the use of the MRC framework coupled with a development of a programme theory, review of existing evidence, initial stakeholder engagement and qualitative research. The resulting intervention was co-developed by a multidisciplinary team, including PPIE members, to explore how existing evidence could be operationalised in practice. This collaborative approach brought together diverse perspectives and professional expertise, contributing to a more comprehensive and contextually relevant intervention than might be achieved by a single discipline alone.

Implementation strategies, which include the provision of the training programme, the intervention manual, and support and supervision to the intervention delivery teams, are typically poorly described in the wider literature [22]. The focus on these strategies in the RecoverED intervention has the potential to contribute to the existing knowledge base and support professionals to deliver delirium, as well as other neurocognitive interventions in a targeted and informed way. Historically, research into enhancing recovery after delirium has been very limited, and this intervention offered the opportunity to evaluate a rehabilitation intervention specifically targeted at this population following hospitalisation.

The complex needs of individuals recovering after delirium necessitate the involvement of a multidisciplinary team, each contributing their expertise. However, blending these diverse perspectives posed a challenge during the design phase, requiring exceptional collaboration to develop cohesion. The intervention needed to be both comprehensive in addressing the various aspects of recovery, and feasible for implementation in real-world clinical settings. This involved careful consideration of the time commitment required from both therapists and RSWs, alongside an awareness of the associated costs.

Another challenge was ensuring meaningful involvement of patients and the public through PPIE, particularly within this population. Achieving a diverse and representative sample proved difficult yet was crucial for ensuring the validity and acceptability of the intervention.

### Implications for research

In line with the MRC framework, the next stage is evaluation of the newly developed intervention. A study was designed to assess the feasibility of a randomised controlled trial [3]. This feasibility study also has an embedded process evaluation, which will provide further insight into the intervention design and implementation (e.g., around fidelity, adherence, acceptability and context) and will be important to understand optimal design of a complex intervention of this type within existing services [23]. The findings from the feasibility study and process evaluation will help further refine the programme theory and logic model by informing our currently held theoretical understanding of post-acute delirium rehabilitation. As the number of people experiencing delirium grows it will be important for research to demonstrate the effectiveness and cost-effectiveness of community-based interventions to ensure that the rehabilitation and support needs of this population are met in a holistic manner.

### Future directions and implementation considerations

There is a lack of clearly described and replicable rehabilitation interventions designed to support recovery after delirium. Evidence suggests that rehabilitation can address impairments in people with long-term, progressive, neurodegenerative conditions, such as dementia [19, 24–27]. This research seeks to apply the theoretical bases for rehabilitation strategies to address similar impairments for people with delirium, who may suffer long-term consequences after discharge from hospital. By focusing on functional recovery, the intervention seeks to help people be more independent, prevent progression of impairments, prevent rehospitalisation, and help people stay in their own homes for longer. This paper presents the various elements of the intervention in a transparent and cohesive way, using the TIDieR tool to provide a thorough description to facilitate replication [15].

Before implications for routine practice can be considered, it is essential to rigorously assess whether the intervention is deliverable, acceptable to patients and staff, and feasible within existing care pathways. A single arm feasibility study with an embedded qualitative process evaluation is currently being undertaken to establish the viability of a definitive trial to evaluate the intervention. Challenges related to undertaking this research, as well as the practical delivery of the intervention outside the research environment, have been explored in related work [28]. Future research using a randomised controlled trial design will be needed to compare the RecoverED intervention with usual care to establish its effectiveness and cost effectiveness, before considering real world implementation.

## Conclusion

This paper has presented the systematic and theory-based development and design of a complex rehabilitation intervention to support recovery following an episode of delirium. In the absence of knowledge and research into treatment after delirium, following hospitalisation, this intervention potentially addresses an important practice gap. Iterative refinements to the intervention will be made in line with the results of the ongoing research.

## Supplementary Information

Below is the link to the electronic supplementary material.

Supplementary Material 1

Supplementary Material 2

Supplementary Material 3

Supplementary Material 4

## Data Availability

The datasets used and/or analysed during the current study are available from the corresponding author on reasonable request.

## Abbreviations

ADL: Activities of Daily Living
CIC: Community Interest Company
CP: Clinical Psychologist
ICF: International Classification of Functioning, Disability and Health
LiFE: Lifestyle-Integrated Functional Exercise
MRC: Medical Research Council
OT: Occupational Therapist
PAL: Pool Activity Level
PPIE: Patient and Public Involvement and Engagement
PT: Physiotherapist
RSW: Rehabilitation Support Worker
SMART: Specific, Measurable, Achievable, Realistic, Time-based
TIDieR: Template for Intervention Description and Replication
