# Supplementary material for: The design and development of a home-based rehabilitation programme for those recovering after an episode of delirium
Source: BMC Health Serv Res. 2025 Nov 12;25:1464. doi: 10.1186/s12913-025-13614-8 (PMC12613552; doi:10.1186/s12913-025-13614-8)
Supplement: Supplementary file 1 — Supplementary Material 1 [file 12913_2025_13614_MOESM1_ESM.docx]

**Supplementary file 1**

Training modules

*Table showing a list of the training modules.*

| **Training module** | **Approx. Length time to complete** |
| --- | --- |
| What is RecoverED? | 10 minutes |
| Delirium | 10 minutes |
| Approaches and Facilitators | 10 minutes |
| Initial Assessment | 20 minutes |
| Planning the intervention | 15 minutes |
| Recovery Record (including psychoeducation, healthy lifestyle guidance, monitoring active treatment, signposting) | 25 minutes |
| Psychosocial recovery | 50 minutes |
| Cognitive recovery | 50 minutes |
| Physical recovery | 35 minutes |
| Completing the CRF | 5 minutes |
| Supervision, halfway review and final session | 10 minutes |
