# Supplementary material for: The design and development of a home-based rehabilitation programme for those recovering after an episode of delirium
Source: BMC Health Serv Res. 2025 Nov 12;25:1464. doi: 10.1186/s12913-025-13614-8 (PMC12613552; doi:10.1186/s12913-025-13614-8)
Supplement: Supplementary file 2 — Supplementary Material 2 [file 12913_2025_13614_MOESM2_ESM.docx]

**Supplementary file 2**

Content of the Recovery Record

*Table showing the content of the Recovery Record.*

| **Recovery Record topic** | **Intended use** |
| --- | --- |
| Delirium information sheet | Developed with a PPIE group this easy to understand information sheet is to support the individuals understanding of delirium and as a resource for support workers and therapists. |
| Goals | Documenting and reviewing SMART goals at each intervention session. For reference between sessions. |
| Health and wellbeing check | Monitoring physical and emotional symptoms. |
| Rehabilitation record | Recording what takes place during each session. As reference for the person and their carer, to aid communication between recipients and intervention delivery team. |
| Diary sheets | Repetition of this orientation activity aims to reorientate the individual. Can be used to record activities between sessions. |
| Recovery timeline | To support individuals to make sense of their delirium experience. |
| Helping with your emotions | Psychosocial recovery |
| Coping with stress | Psychosocial recovery |
| Coping with worry | Psychosocial recovery |
| Three good things | Psychosocial recovery |
| Grounding exercise | Psychosocial recovery |
| Calming your breathing | Psychosocial recovery |
| Memory | Cognitive recovery |
| Sleep | Healthy lifestyle advice |
| Food and drink | Healthy lifestyle advice |
| Your home and your recovery | Cognitive recovery resource aimed at optimising the therapeutic environment. |
| Physical activity guidance | Physical recovery, personalised where appropriate. |
| Signposting and onwards referral | A record of any identified signposting or onward referral activity for either member of the dyad. |
| Activity record sheet | A record of the personalised physical recovery activities completed. This can then be reviewed at the weekly visits. |
