# Supplementary material for: The design and development of a home-based rehabilitation programme for those recovering after an episode of delirium
Source: BMC Health Serv Res. 2025 Nov 12;25:1464. doi: 10.1186/s12913-025-13614-8 (PMC12613552; doi:10.1186/s12913-025-13614-8)
Supplement: Supplementary file 3 — Supplementary Material 3 [file 12913_2025_13614_MOESM3_ESM.docx]

**Supplementary file 3**

Adapted Activity Profile

*Activity level guidance sheets – three different levels.*
