# Supplementary material for: The design and development of a home-based rehabilitation programme for those recovering after an episode of delirium
Source: BMC Health Serv Res. 2025 Nov 12;25:1464. doi: 10.1186/s12913-025-13614-8 (PMC12613552; doi:10.1186/s12913-025-13614-8)
Supplement: Supplementary file 4 — Supplementary Material 4 [file 12913_2025_13614_MOESM4_ESM.docx]

**Supplementary file 4**

**Case study - Mr. X** ​

- ​Mr. X is a 78-year-old gentleman who lives with his wife. Admitted to the acute hospital with confusion and poor mobility. Treated for a urinary tract infection and diagnosed with delirium. ​
- No prior diagnosis of dementia. No concerns with mental capacity. Reports of mild cognitive impairment since a stroke 4 years prior. No other significant medical history. ​
- Remained in hospital for 14 days before discharge home. Discharged home with no package of care.​

**Initial assessment and intervention planning**​

- Initial assessment completed at home by a physiotherapist, with his wife present. ​
- Participation goal set by the participant; ‘ **To return to playing golf within 3 months.’**​
- Identified that the participant is very motivated to regain his previous physical level as he has a strong driver to return to playing golf. ​
- Activities working on balance were chosen as key to helping him achieve this goal, as well as increasing stamina with outdoor mobility.​

**Management plan**

- The RSW monitored recovery from the infection (monitoring active treatment), responded to carers questions about delirium (delirium education) and offered some guidance on nutrition to support recovery (healthy lifestyle guidance) throughout the course of intervention delivery.

**Physical recovery**

- To return to playing golf Mr. X needed to improve his balance and his stamina. A selection of activities were selected to help support this element of his recovery. They were completed in the kitchen whilst waiting for the kettle to boil, for example. Mrs X encouraged and supervised with these in between intervention sessions initially, but he then became independent with them. ​
- Stamina was also considered important for his goal attainment, so intervention sessions worked on outdoor mobility, gradually increasing the distance walked. ​

**Review​**

- At the halfway point Mr. X was visited by the RSW and the physiotherapist. ​
- Goals were reviewed and updated as appropriate in collaboration with Mr. and Mrs. X (person centered care and tailoring).
- He was making good progress towards his physical goal. The physiotherapist progressed the difficulty level for the balance activities and increased the outdoor mobility distance (tailoring). ​
- Cognitive and psychosocial needs were identified at this stage, as a result new goals were made and additional elements to the intervention were introduced as described below.

**Cognitive recovery**

- Mr. X’s goal was based around a physical recovery need. No cognitive deficits were identified on assessment and input around psychosocial recovery was not seen as required. However, as the sessions progressed other needs were identified. ​
- Whilst practicing outdoor mobility the RSW noticed that Mr. X couldn’t plan a route and had poor safety awareness when crossing the road.
- The RSW used a flexible approach, in discussion with the occupational therapist and brought in some cognitive recovery components during subsequent sessions. ​
- They looked at sequencing and planning; considering what clothes and equipment were needed before he went out. They worked on route planning whilst completing outdoor mobility practice, considering risk and safety. ​
- The timeline activity was also used to support memory. ​

**Psychosocial recovery**

- No need for any psychosocial recovery input was identified initially. ​
- During a couple of sessions, the RSW noted some irritation and frustration from Mr. X. His wife also reported a couple of episodes where he had become verbally aggressive with her. ​
- The RSW introduced the 5,4,3,2,1 technique to ground Mr. X and bring his focus back to the present moment. ​
- The other resources in the recovery record were used by Mrs. X, she found the education documents reassuring and a useful tool to refer to. ​The RSW spent some time with Mrs. X to ensure her needs were also considered (carer support and involvement).

**Discharge​**

- The participant received 10 sessions, all completed face to face. ​
- Upon discharge he had almost achieved his goal. ​
- He was happy to continue with his activity plan with the support of his wife. The couple were referred to the community nursing team for ongoing management of some physical health needs. ​

**Goals**

| Participation goal | **To return to playing golf within 3 months.** |
| --- | --- |
| Updated Participation goal (after review) | **To return to playing a full round of golf, once a week in 6 weeks' time.** |
| Cognitive recovery goal | **None set originally – not seen as required** |
| Updated cognitive recovery goal (after review) | **To be able to plan a safe and appropriate route for my daily walk within 2 weeks.**​ |
| Physical recovery goal | **To improve my balance so that I feel steady enough to practice my golf swing in 2 weeks' time.** ​ |
| Updated physical recovery goal | **To be able to walk to the closest post box and back, each day , in 3 weeks' time.**​ |
| Psychosocial recovery goal | **None set originally – not seen as required** |
| Updated psychosocial recovery goal | **Practice techniques so that I can use them independently to manage feelings of frustration or irritation should they arise.​** |
